# Discrepancy between true ankle dorsiflexion and gait kinematics and its association with severity of planovalgus foot deformity

**DOI:** 10.1186/s12891-020-03285-3

**Published:** 2020-04-16

**Authors:** Ki Hyuk Sung, Chin Youb Chung, Kyoung Min Lee, Ki Bum Kwon, Jeong Hyun Lee, Moon Seok Park

**Affiliations:** Department of Orthopaedic Surgery, Seoul National University Bundang Hospital, 82 Gumi-ro 173 Beon-gil, Bundang-Gu, Sungnam, Gyeonggi 13620 South Korea

**Keywords:** Planovalgus, Ankle dorsiflexion, Physical examination, 3-dimensional gait analysis

## Abstract

**Background:**

In planovalgus deformity with triceps contracture, a midfoot break happens, and ankle dorsiflexion (ADF) occurs at the mid-tarsal joint during gait. Results of standard 3D gait analysis may misinterpret the true ankle dorsiflexion because it recognizes the entire foot as a single rigid segment. We performed this study to investigate whether the severity of planovalgus deformity is associated with the discrepancy between the value of ADF evaluated by physical examination and 3-dimensional (3D) gait analysis. In addition, we aimed to identify the radiographic parameters associated with this discrepancy and their relationships.

**Methods:**

Consecutive 40 patients with 65 limbs (mean age, 11.7 ± 5.5 years) with planovalgus foot deformity and triceps surae contracture were included. All patients underwent 3D gait analysis, and weightbearing anteroposterior (AP) and lateral (LAT) foot radiographs. ADF with knee extension was measured using a goniometer with the patient’s foot in an inverted position.

**Results:**

Twenty-one limbs underwent operation for planovalgus foot deformity, and 56 limbs underwent operation for equinus deformity. The difference between ADF on physical examination and ADF at initial contact on gait analysis was 17.5 ± 8.4°. Differences between ADF on physical examination and ADF at initial contact on gait analysis were significantly associated with the LAT talus-first metatarsal angle (*p* = 0.008) and calcaneal pitch angle (*p* = 0.006), but not associated with the AP talus-first metatarsal angle (*p* = 0.113), talonavicular coverage angle (*p* = 0.190), talocalcaneal angle (*p* = 0.946), and naviculocuboid overlap (*p* = 0.136).

**Conclusion:**

The discrepancy between ADF on physical examination and 3D gait analysis was associated with the severity of planovalgus deformity, which was evaluated on weightbearing LAT foot radiographs. Therefore, physicians should be cautious about interpreting results from 3D gait analysis and perform a careful physical examination to assess the degree of equinus deformity in patients with planovalgus foot deformity.

## Background

Planovalgus deformity is a common foot problem characterized by hind foot valgus, midfoot pronation, decreased medial longitudinal arch, and forefoot abduction/supination [1, 2]. In the flexible type, the medial longitudinal arch is restored performing the Jack and tiptoe tests, and heel alignment is restored when standing on tiptoes. However, the medial arch is not restored in the tiptoe test due to the restricted motion of the subtalar joint in the rigid type, which is often associated with tarsal coalition [3, 4]. Planovalgus is often associated with equinus deformity in patients with cerebral palsy or an idiopathic cause due to contracture of the triceps surae. Most patients are asymptomatic and do not require treatment. However, planovalgus deformity with triceps surae contracture in contrast to simple flexible planovalgus is known to often be symptomatic and cause pain, medial foot callosity, and functional disability [4, 5]. In addition, a previous study using gait analysis showed that increased forefoot abduction occurred throughout the stance phase in symptomatic feet compared to asymptomatic feet [6]. When the deformity becomes symptomatic and conservative treatment fails, operative treatment including calcaneal osteotomy, arthroereisis, talonavicular fusion, or triple arthrodesis can be considered [2, 4, 7, 8]. For patients with contracture of the triceps surae, triceps surae lengthening procedures, such as tendo-Achilles lengthening and the Strayer procedure, should be performed concomitantly.

For patients with planovalgus deformity, general examination of the musculoskeletal system including foot and ankle joints should be performed, especially in patients with cerebral palsy. A physical examination or 3-dimensional (3D) gait analysis can be used to evaluate the degree of equinus deformity. In planovalgus deformity with triceps contracture, a midfoot break happens, and ankle dorsiflexion occurs at the mid-tarsal joint during gait. Therefore, the degree of ankle dorsiflexion (ADF) should be assessed with the subtalar joint in neutral position for mid-tarsal joint locking to prevent dorsiflexion at the mid-tarsal joint during the physical examination [4]. However, results of standard 3D gait analysis may misinterpret the true ankle dorsiflexion because it recognizes the entire foot as a single rigid segment [9]. Therefore, physicians should be cautious about interpreting results from 3D gait analysis when assessing the degree of ADF in patients with planovalgus foot deformity.

We hypothesized that the discrepancy between ADF on physical examination and 3D gait analysis would be associated with the severity of planovalgus deformity, which can be evaluated by plain radiographs. We performed this retrospective cohort study to confirm our hypothesis. In addition, we identified the radiographic parameters associated with this discrepancy and their relationships.

## Materials and methods

This study was approved by our hospital’s institutional review board, and the need for informed consent was waived because of the retrospective nature of this study.

### Study population and data collection

The inclusion criteria were as follows: (1) consecutive patients who visited our hospital for symptomatic planovalgus foot deformity with triceps surae contracture between 2016 and 2018, (2) patients who had weight-bearing anteroposterior (AP) and lateral (LAT) foot radiographs, and (3) patients who underwent 3D gait analysis. The exclusion criteria were as follows: (1) patients who had inadequate foot radiographs for measurements or gait analysis data, (2) patients who had previous surgery for equinus or foot deformity at the time of data collection, and (3) patients who received botulinum toxin injection within 6 months.

From the patients’ medical record review, we collected information regarding patients’ age, sex, etiology, and operative procedures.

### Radiographic parameters

**Fig. 1:**
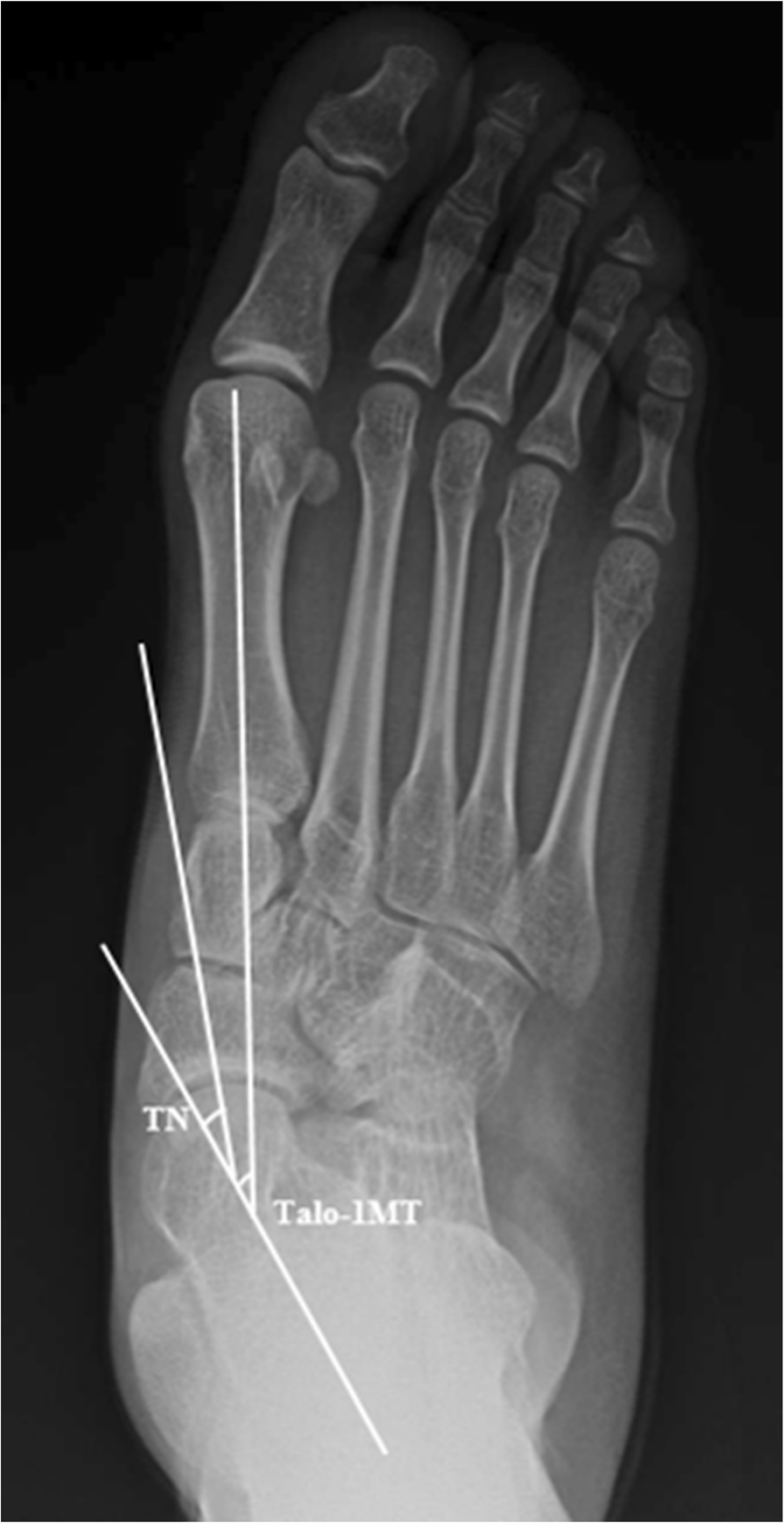
On weightbearing anteroposterior (AP) foot radiographs, the AP talus-first metatarsal angle (AP talo-1MT) and talonavicular coverage angle (TN) are measured

Radiographic parameters for assessing the severity of planovalgus deformity were selected based on literature reviews [2, 10–13]. On weightbearing AP foot radiographs, the AP talus-first metatarsal angle (AP talo-1MT) and talonavicular coverage angle (TN) were measured (Fig. 1). AP talo-1MT was defined as the angle between a line drawn through the midpoints of the talar head and neck, and a line bisecting the long axis of the first metatarsal bone. TN was defined as the angle between a line drawn through the midpoints of the talar head and neck, and a line bisecting the proximal articular surface of the navicula.

**Fig. 2:**
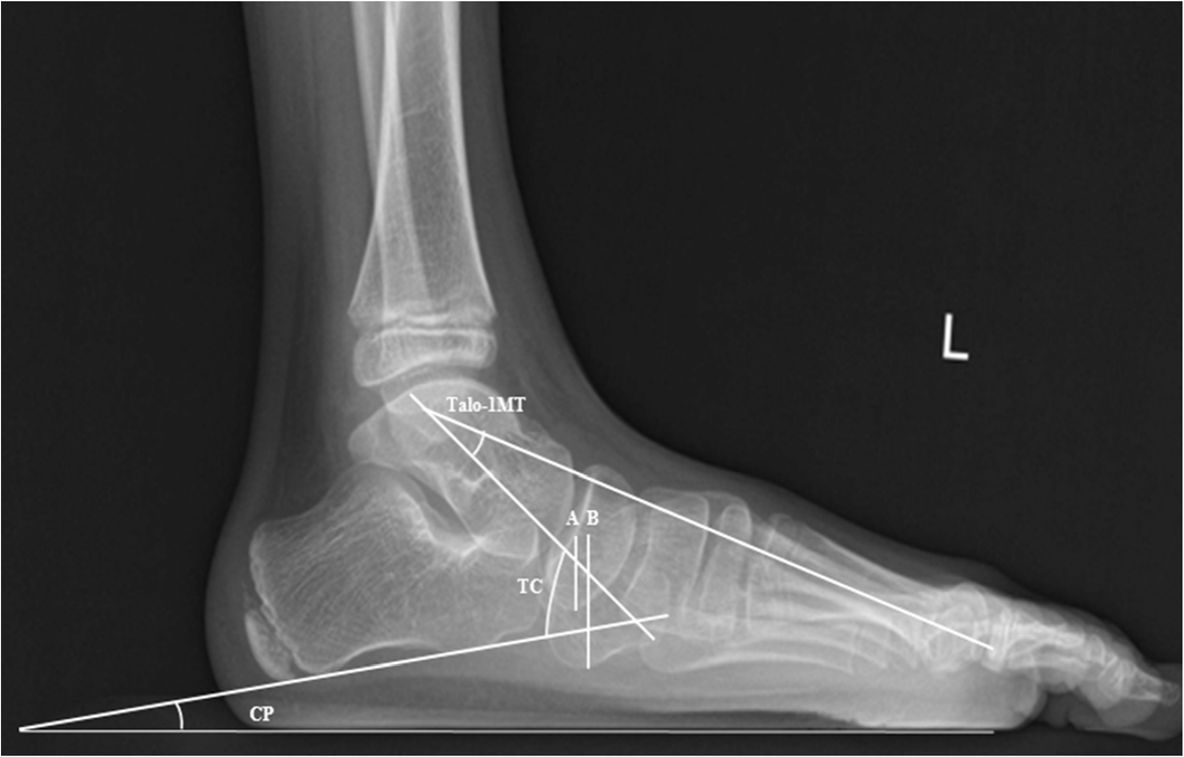
On weightbearing lateral (LAT) foot radiographs, the LAT talus-first metatarsal angle (LAT talo-1MT), calcaneal pitch angle (CP), LAT talocalcaneal angle (TC), and naviculocuboid (NC) overlap are measured

On weightbearing LAT foot radiographs, the LAT talus-first metatarsal angle (LAT talo-1MT), calcaneal pitch angle (CP), LAT talocalcaneal angle (TC), and naviculocuboid (NC) overlap were measured (Fig. 2). LAT talo-1MT was defined as the angle between a line bisecting the long axis of the first metatarsal bone and a line drawn through the midpoints of the talar head and neck. CP was defined as the angle between a line drawn along the edge of the plantar soft tissue shadow and a line drawn along the lower margin of the calcaneus. TC was defined as the angle between a line drawn through the midpoints of the talar head and neck, and a line drawn along the lower margin of the calcaneus. NC overlap was defined as the overlapping portion of the navicula and cuboid divided by the vertical height of the cuboid.

Radiographic measurements were performed using picture archiving and communication software (INFINITT Healthcare, Seoul, Korea). Before the main measurements were determined, the inter-observer reliability of the radiographic measurements of 15 AP and LAT standing foot radiographs was tested by two orthopedic surgeons (KBK and JHL). Thereafter, radiographic measurements for all patients were determined by one author (JHL).

### Gait analysis

All patients underwent 3D gait analysis using a Motion Analysis system (Motion Analysis Corporation, CA, USA) that was equipped with 10 cameras and two force plates. Markers were placed by two operators according to the Helen Hayes marker set. Nineteen markers were used in the static trials, and 15 lower body markers were used in the dynamic walking trials [14]. Patients walked barefoot on a 9-m walkway over three times at an interval of approximately 30 s, and kinematic data and temporal parameters were recorded. Three trials representing patients’ typical gait pattern were selected and averaged. Sagittal kinematics of the ankle joint including ankle dorsiflexion at initial contact, mean ankle dorsiflexion in the stance phase, and peak ankle dorsiflexion in the stance phase were considered as outcome variables.

### Physical examination

Physical examinations were performed by pediatric orthopedic fellows, before 3D gait analysis, and a goniometer was used to assess range of motions of the hip, knee, and ankle joints. For the ankle joint, one arm of the goniometer was placed along a bisection of the LAT aspect of the lower leg, and the other arm was placed along the LAT margin of the hindfoot. When measuring ADF, the foot was inverted to lock the mid-tarsal joint and to avoid dorsiflexion at the mid-tarsal joint. ADF with knee extension was considered as an outcome variable.

### Statistical analysis

Intraclass correlation coefficients (ICCs) and 95% confidence intervals were used to assess interobserver reliabilities for radiographic measurements. ICCs were calculated in the setting of a two-way random effect model, assuming a single measurement and absolute agreement [15]. ICC values > 0.8 indicate excellent reliabilities.

Descriptive statistics, such as means and standard deviations, were used to summarize the data. To consider the inclusion of bilateral limbs, a linear mixed model was used to estimate the discrepancy between ADF on physical examination and 3D gait analysis with radiographic parameters [16].

Statistical analyses were performed using SAS 9.4 (SAS Institute, Cary, NC, USA) and SPSS software for Windows (version 25.0; IBM Corp., Armonk, NY, USA). All statistics were two-tailed, and a *p*-value < 0.05 was considered significant.

## Results

Fourty-four patients with 73 limbs were initially identified in our database. After applying the exclusion criteria, 40 patients with 65 limbs were finally included in this study. Etiologies were cerebral palsy in 33 patients and idiopathic in 7 patients. Mean patients’ age at the time of evaluation was 11.7 years (median, 9.5; range, 5.2–25.3) and 35 patients were under 18 years old. Bilateral feet were included in 25 patients and unilateral foot in 15 patients. No patients showed arthritic change of the ankle joint. Among the 40 patients, 36 patients with 58 limbs underwent surgeries. The total number of surgical procedures was 158 (2.7 per limbs). Twenty-one limbs underwent operation for planovalgus foot deformity (calcaneal lengthening, 20; talonavicular fusion, 1), and 56 limbs underwent operation for equinus deformity (Table 1).

**Table 1:** Patient demographics and summary of operative procedures

Parameters	Values
Sex (male / female)	29 / 11
Age (years)	11.7 ± 5.5 (range: 5.2–25.3)
Side (right / left)	35 / 30
Etiology (Cerebral palsy / Idiopathic)	33 / 7
Operative procedures	Number of limbs
Calcaneal lengthening	20
Talonavicular fusion	1
Intramuscular psoas lengthening	5
Adductor tenotomy	2
Dega pelvic osteotomy	2
Femoral derotaton osteotomy	7
Femoral varization osteotomy	2
Distal hamstring lengthening	32
Rectus femoris transfer	27
Tendo-Achilles lengthening or Strayer procedure	56
Tibial derotation osteotomy	2
Kidner operation	2

Radiographic measurements had excellent interobserver reliabilities for all radiographic parameters (ICC, 0.860–0.985) (Table 2). On physical examination, ankle dorsiflexion in knee extension was − 20.6 ± 11.3°. On sagittal kinematics of gait analysis, ADF at initial contact, mean ADF in the stance phase and peak ADF in the stance phase were − 3.2 ± 7.0°, 5.2 ± 7.5° and 11.3 ± 8.5°, respectively (Table 3). The difference between ADF on physical examination and ADF at initial contact on gait analysis was 17.5 ± 8.4°.

**Table 2:** Inter-observer reliabilities of radiographic measurements

	Inter-observer reliability	
	ICC	95% CI
AP talus-1st metatarsal angle	0.976	0.930–0.992
Talonavicular coverage angle	0.876	0.549–0.961
Lateral talus-1st metatarsal angle	0.969	0.908–0.990
Calcaneal pitch angle	0.985	0.954–0.995
Talocalcaneal angle	0.918	0.779–0.971
Naviculocuboid overlap	0.981	0.945–0.994

*ICC* intraclass correlation coefficient, *CI* confidence interval

**Table 3:** Summary of radiographic measurements, physical examination and sagittal kinematics of ankle joint

	Mean ± SD	Range
Radiographic parameter		
AP talus-1st metatarsal angle (°)	23.5 ± 13.7	0.7 to 73.4
Talonavicular coverage angle (°)	32.3 ± 12.3	8.5 to 61.9
Lateral talus-1st metatarsal angle (°)	33.9 ± 15.1	8.1 to 76.8
Calcaneal pitch angle (°)	8.9 ± 7.6	−14.4 to 21.7
Talocalcaneal angle (°)	56.1 ± 9.1	30.9 to 77.3
Naviculocuboid overlap (%)	75.6 ± 19.2	13.0 to 100
Physical examination		
Ankle dorsiflexion in knee extension (°)	−20.6 ± 11.3	−50.0 to −5.0
Sagittal kinematics of ankle joint		
Ankle dorsiflexion at initial contact (°)	−3.2 ± 7.0	−36.5 to 8.7
Mean ankle dorsiflexion in stance (°)	5.2 ± 7.5	− 37.7 to 19.4
Peak ankle dorsiflexion in stance (°)	11.3 ± 8.5	− 32.8 to 29.8

**Fig. 3:**
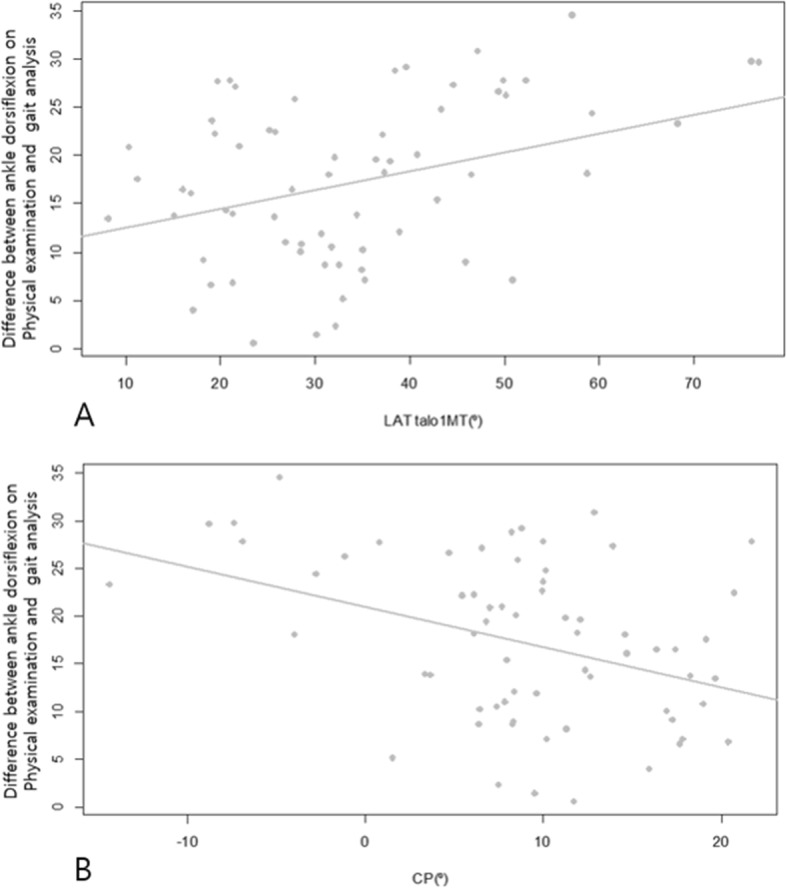
Scatter plot showing the linear relationship (**a**) between the lateral talus-first metatarsal angle (LAT talo-1MT) and differences between ankle dorsiflexion on physical examination and ankle dorsiflexion initial contact on gait analysis, and (**b**) between calcaneal pitch angle (CP) and differences between ankle dorsiflexion on physical examination and ankle dorsiflexion initial contact on gait analysis

Differences between the ADF on physical examination and the ADF at initial contact on gait analysis were significantly associated with LAT talo-1MT (*p* = 0.008) and CP (*p* = 0.006) (Fig. 3a and b), but not with AP talo-1MT (*p* = 0.113), TN (*p* = 0.190), TC (*p* = 0.946), and NC overlap (*p* = 0.136). Similar findings were found in terms of differences between ADF on physical examination and mean ADF in the stance phase, and between ADF on physical examination and peak ADF in the stance phase (Table 4).

**Table 4:** Associated radiographic parameters with the difference between ankle dorsiflexion on physical examination and gait analysis

	ADF at initial contact - ADF			Mean ADF in stance - ADF			Peak ADF in stance - ADF		
	Estimate	SE	*p*-value	Estimate	SE	*p*-value	Estimate	SE	*p*-value
AP talus-1st metatarsal angle	−0.13	0.08	0.113	−0.12	0.09	0.203	−0.10	0.10	0.353
Talonavicular coverage angle	−0.14	0.08	0.190	−0.193	0.09	0.052	−0.19	0.10	0.068
Lateral talus-1st metatarsal angle	−0.19	0.07	0.008	−0.22	0.08	0.010	−0.23	0.08	0.011
Calcaneal pitch angle	0.42	0.14	0.006	0.41	0.17	0.023	0.44	0.18	0.023
Talocalcaneal angle	0.01	0.11	0.946	−0.01	0.13	0.917	−0.03	0.14	0.837
Naviculocuboid overlap	8.47	5.49	0.136	8.56	6.41	0.195	8.63	6.86	0.220

*ADF* ankle dorsiflexion

## Discussion

We demonstrated that the discrepancy between ADF assessed by physical examination and 3D gait analysis is significantly associated with two radiographic parameters on weight-bearing LAT foot radiographs: LAT talo-1MT and CP. This finding supports our hypothesis that the discrepancy between ADF on physical examination and 3D gait analysis is associated with the severity of planovalgus deformity.

ADF is essential for the forward translation of the center of gravity of the body during the second rockers of the gait cycle [17]. If the ADF is restricted due to the contracture of the triceps surae, ADF occurs at the mid-tarsal joint, and this may eventually lead to midfoot breakage and planovalgus foot deformity. For patients with planovalgus deformity, it is essential to evaluate the degree of ADF in order to determine appropriate treatment methods.

In the standard 3D gait analysis, the rigid single-segment foot model is based on a vector that passes from the calcaneus to the space between the 2nd and the 3rd metatarsal. The motion of this foot vector relative to the plantar surface represents the ankle plantarflexion and dorsiflexion [18]. When the patient presents with a midfoot breakage, this marker placement can result in the exaggeration of ADF. In other words, an insufficient amount of ADF is compensated by midfoot breakage in a planovalgus deformity [9]. To avoid or reduce this measurement artifact, a toe marker can be placed more proximally toward the midfoot by the clinician or the operators in gait lab [19]. However, this toe marker adjustment was not made in this study due to its retrospective design.

To overcome the disadvantage of standard 3D gait analysis, 3D multi-segment foot models, such as the DuPont foot model, Oxford foot model, Milwaukee foot model, Leardini foot model, and Heidelberg foot model, have been increasingly used because they can illustrate the effect of foot and ankle pathologies on intersegmental motion of the foot [20–27]. However, repeatability of the multi-segment foot model is lower in the pediatric population than in the adult population because it is difficult to attach the marker to the correct position in small feet [28, 29]. Thus, use of the 3D multi-segment foot model might be limited in pediatric patients. Therefore, a careful physical examination should be performed to assess ankle range of motion in planovalgus deformity. The subtalar joint must be inverted to the neutral position and locked in that position in order to isolate and evaluate the dorsiflexion at the ankle joint, not the mid-tarsal joint.

We think that there may be a discrepancy between true ADF on physical examination and ADF on gait analysis, and that the severity of planovalgus deformity may be associated with this discrepancy. The severity of planovalgus deformity can be evaluated on weight-bearing AP and LAT foot radiographs. Previous studies showed that AP talo-1MT, TN, NC overlap and LAT talo-1MT were valid and reliable radiographic indices for evaluating planovalgus foot deformity [2, 11]. This study also showed that these radiographic indices had excellent interobserver reliabilities for measurements indicating their relevance for clinical use. Other radiographic parameters including Kite’s angle, Costa-Bertani’s angle, and the talar declination angle also can be used to evaluate planovalgus deformity [30]. In this study, only two radiographic parameters including LAT talo-1MT and CP, which were measured on LAT radiographs, were associated with the discrepancy between ADF on physical examination and 3D gait analysis. However, radiographic parameters on AP radiographs including AP talo-1MT and TN were not associated with this discrepancy. LAT talo-1MT is the angle between the longitudinal axis of the first metatarsal bone and talus on a LAT weightbearing foot radiograph. Increased LAT talo-1MT indicates a more vertically positioned talus, which represents increased midfoot breakage and increased dorsiflexion at the mid-tarsal joint. CP represents the relative position of the calcaneus to the ground, and decreased CP means increased dorsiflexion at the mid-tarsal joint. Our finding that increased LAT talo-1MT and decreased CP were associated with the discrepancy between ADF on physical examination and 3D gait analysis is reasonable. Therefore, clinicians should consider LAT talo-1MT and CP to assess ADF from 3D gait analysis in patients with planovalgus deformity.

Contracture of the triceps surae lead to midfoot breakage and planovalgus foot deformity. Correction of the equinus deformity by tendo-Achilles lengthening might help ameliorate planovalgus deformity as improvement in dorsiflexion of the ankle joint results in the improvement of midfoot breakage. Further study regarding the changes in bony alignment of the foot after tendo-Achilles lengthening for planovalgus deformity is required.

There are limitations to this study. First, we used a goniometer to measure ADF despite lower reported reliability values with use of this device. Several authors introduced various valid and reliable devices for measuring ADF [31–33]. However, other devices could not measure ADF with the foot in an inverted position. In addition, the goniometer is commonly used in the clinical setting, and ADF was measured using a uniform protocol in this study. Therefore, we think that our results may be clinically meaningful. Second, our results may not be generalized to all patients with planovalgus deformities and triceps surae contractures, because most of our study subjects were children and adolescents. Therefore, further study on an adult cohort is required. Third, ADF with knee extension was considered an outcome variable in this study. Use of ADF with knee flexion might change the results. However, the results regarding ADF with knee flexion are absent due to the retrospective design of this study. Therefore, further prospective research including ADF with both knee extension and knee flexion is needed.

## Conclusions

The discrepancy between ADF on physical examination and 3D gait analysis was associated with the severity of planovalgus deformity, which was evaluated on weightbearing LAT foot radiographs. Therefore, physicians should be cautious about interpreting results from 3D gait analysis and perform a careful physical examination to assess the degree of equinus deformity in patients with planovalgus foot deformity. Further research is required to develop and validate sophisticated foot marker placements when performing standard 3D gait analysis in patients with foot deformity.

## Data Availability

The datasets used and/or analysed during the current study available from the corresponding author on reasonable request.

## Abbreviations

3D: 3-dimensional
ADF: Ankle dorsiflexion
AP: Anteroposterior
LAT: Lateral
talo-1MT: Talus-first metatarsal angle
TN: Talonavicular coverage angle
CP: Calcaneal pitch angle
TC: Lateral talocalcaneal angle
NC: Naviculocuboid
ICC: Intraclass correlation coefficient
